# CM-AVM syndrome in a neonate: case report and treatment with a novel flow reduction strategy

**DOI:** 10.1186/2045-824X-4-19

**Published:** 2012-11-20

**Authors:** Gerald G Behr, Leonardo Liberman, Jocelyn Compton, Maria C Garzon, Kimberly D Morel, Christine T Lauren, Thomas J Starc, Stephen J Kovacs, Vincent Beltroni, Rachel Landres, Kwame Anyane-Yeboa, Philip M Meyers, Emile Bacha, Jessica J Kandel

**Affiliations:** 1 Department of Radiology, Division of Pediatric Radiology Columbia University, New York, NY, USA; 2 Department of Pediatrics, Division of Cardiology Columbia University, New York, NY, USA; 3 Columbia College of Physicians & Surgeons, New York, NY, USA; 4 Department of Dermatology and Pediatrics, Columbia University, New York, NY, USA; 5 Department of Pediatrics, Division of Clinical Genetics, Columbia University, New York, NY, USA; 6 Department of Radiology, Division of Interventional Neuroradiology, Columbia University, New York, NY, USA; 7 Department of Surgery, Division of Cardiothoracic Surgery, Columbia University, New York, NY, USA; 8 Department of Surgery, Division of Pediatric Surgery, Columbia University, New York, NY, USA; 9 Division of Neonatology, Vassar Brothers Medical Center, Poughkeepsie, NY, USA

**Keywords:** CM-AVM, RASA 1, Parkes-Weber, Port wine stain, Vascular malformation

## Abstract

Mutations in the RASA-1 gene underlie several related disorders of vasculogenesis. Capillary malformation-arteriovenous malformation (CM-AVM) is one such entity and was recently encountered in a neonate who demonstrated its clinical and radiologic features. A single mutation in the RASA-1 gene was detected.

A novel flow reduction strategy was employed to a large AVM affecting the patient’s upper limb. The imaging findings, surgical procedure and patient’s improved post-operative state are described.

## Background

RASA-1 gene mutations present with a variety of clinical phenotypes. Parkes-Weber syndrome (PKWS) and the more recently described capillary malformation - arteriovenous malformation syndrome (CM-AVM) are two such entities. The hallmarks of both PKWS and CM-AVM are the presence of fast-flow vascular malformations and capillary malformations (CM). A “fast-flow” vascular malformation is defined as a lesion containing abnormal fistulous connections between arteries and veins, without an intervening capillary bed. This includes both arteriovenous malformations (AVM) and arterio-venous fistula (AVF).

Capillary malformations are present in 0.3% of neonates, and appear as a characteristic pink macular stain, often described as a "port wine stain" [1]. Histologically, the lesion is characterized by wide caliber or increased number of dermal capillaries [2]. Although CMs are usually isolated, they may arise in the setting of a syndrome, such as Klippel-Trenaunay, Sturge-Weber, or Proteus. If capillary malformations are associated with a syndrome that incorporates a high-flow lesion such as PKWS or CM-AVM, high output heart failure may ensue. In these instances, most commonly only the CM is evident at birth, and the AVM or AVF presents later in childhood.

In this report, we present a neonate diagnosed at birth with a large upper extremity capillary malformation and a clinically apparent fast-flow vascular malformation with limb enlargement, in the context of a strong family history of vascular malformations and highlight the radiologic characteristics.

## Case presentation

The patient was born at an outside hospital at 41 weeks gestation via spontaneous vaginal delivery to a 20 year-old G2P2. Birth weight was 3991 g and head circumference was 36 cm (both values above the 95 percentile). The pregnancy and delivery were reportedly without complication. The infant was transferred to our institution on the fourth day of life for further management of a vascular skin lesion and upper extremity enlargement and edema.

Upon questioning, there was a family history of vascular birthmarks. The patient's mother has several pink macules on her trunk and extremities. An older sibling also exhibited a port wine stain.

**Figure 1:**
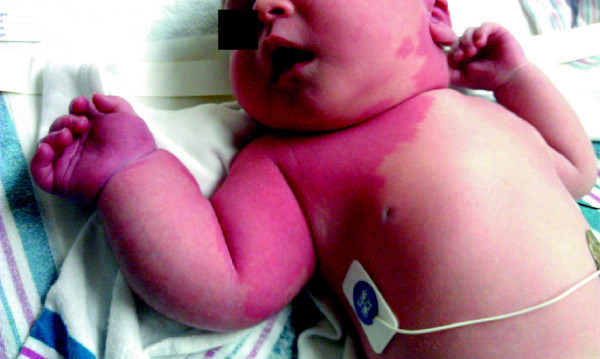
**First day of life:** There is a macular rash extending from the left neck, across the chest and involving the right upper extremity which is larger than the left upper extremity

The neonate was hemodynamically stable. On physical examination, there were blanching salmon-colored macules, extending over the left cheek, left ear, chin and across the left neck (Figure 1). There were darker, pink/violaceous lesions over the left temporal and occipital scalp. A pink patch extended over most of the right upper extremity, intermingled with areas that appeared more violaceous. The entire right upper extremity was enlarged and tensely edematous. The right arm measured 17 cm in length, 1 cm longer than the right, and the right lower arm circumference measured 14.5 cm, 3.5 cm greater than the left. Axillary, brachial, and radial pulses were bounding. At the initial and subsequent examinations, the baby was noted to move the right arm only minimally as compared with the left. The remainder of the physical examination was unremarkable.

There was no laboratory evidence of coagulopathy or platelet consumption.

### Imaging studies

**Figure 2:**
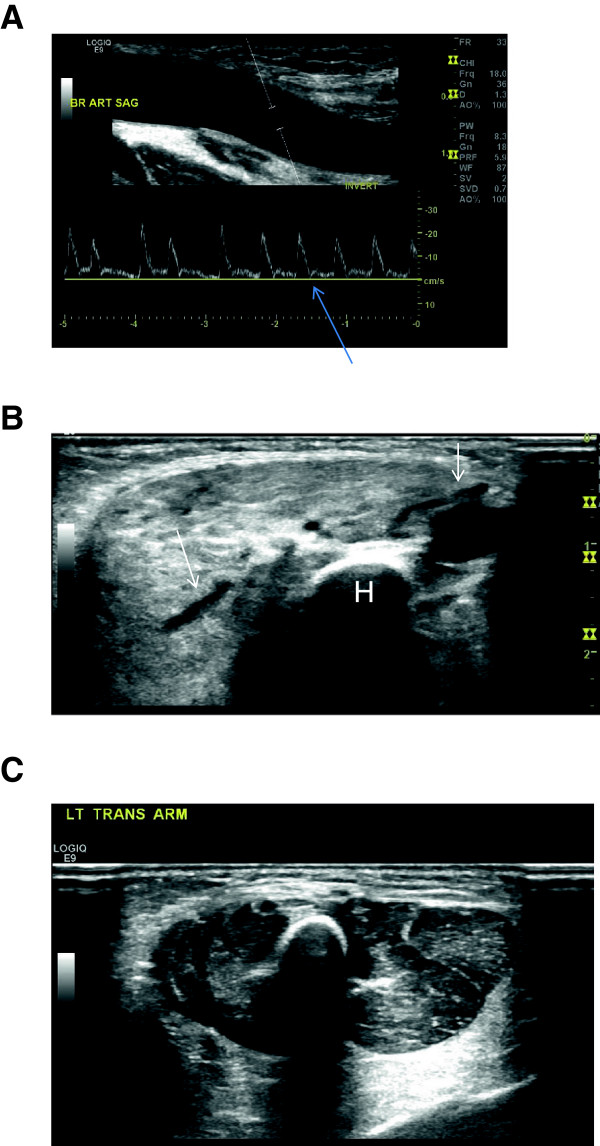
**Ultrasound:** 2**A**) Doppler waveform obtained from the right brachial artery reveals absence of an expected transient reversal of flow (arrow). Also seen is massive dilation of the brachial artery (calipers in artery). 2**B**) Dilated vessels (arrows) are seen coursing through the abnormally hyperechoic, poorly defined and enlarged musculature. Humerus (H). 2**C**) Normal left arm for comparison

Prenatal screening ultrasound was reportedly normal. An echocardiogram performed at the referring institution on the first day of life was also reportedly normal. Ultrasound performed on the fourth day of life revealed massive dilation of the right subclavian, axillary and brachial artery (Figure 2A) as well as a dilated deep venous system. The diameter of the brachial artery was 5 mm. Spectral Doppler analysis of the affected arteries demonstrated a low resistance waveform (Figure 2A). There was arterialization of the waveforms obtained from the adjacent draining veins. There was increased vascularity of the forearm. Additionally, the musculature appeared homogeneously hyperechoic; however, there was no discrete mass (Figure 2B).

**Figure 3:**
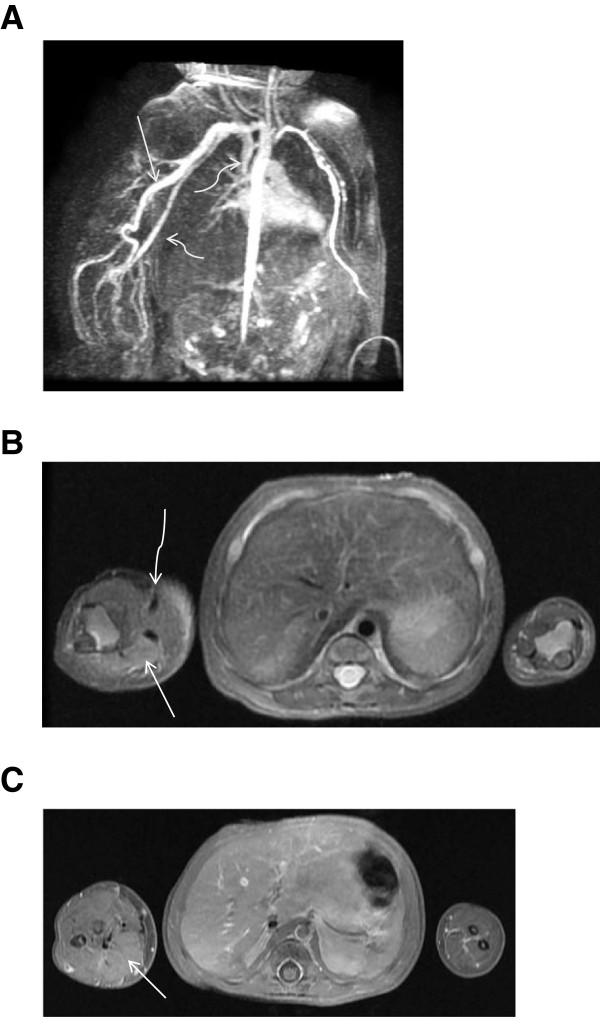
**MRI 3A)Time-resolved MR angiogram shows near simultaneous opacification of a markedly dilated right upper extremity artery(arrow)and vein(curved arrows).** 3**B**) T2 weighted axial sequence shows increased signal and enlargement of right upper extremity soft tissues (arrow) and large vascular flow voids (curved arrow). 3**C**) T1 fat saturated post-gadolinium axial sequence suggests enhancement of the musculature in the right forearm (arrow) compared with the left forearm.

An MRI of the right upper extremity and chest obtained on the seventh day revealed the aneurysmal vessels, extending from the origin of the right subclavian artery to the right brachial artery. Time-resolved post contrast magnetic resonance angiography (TRICKS, General Electric) showed near-simultaneous enhancement of the right upper extremity arteries and draining dilated veins (Figure 3A). There was abnormally high T2 signal in the musculature, though no mass was identified (Figure 3B). An MRI brain scan and a chest radiograph were both normal.

A postnatal echocardiogram was interpreted as mild right ventricular hypertrophy with normal biventricular function. The superior vena cava return to the right atrium appeared increased, consistent with increased flow. The right subclavian artery was markedly dilated.

Taken together, these are consistent with high-grade arteriovenous shunting in the right arm, with resultant massive dilatation of arteries and veins with evidence of increased cardiac output.

### Genetic testing

Sequencing of the RASA-1 coding region revealed a single mutation (c.2603 + 1 G > A one copy).

### Management

The Columbia University Vascular Anomalies Group was consulted. Due to the appearance of the swollen arm, and the dramatic imaging findings, the patient was considered to be at risk for heart failure, vascular "steal" phenomenon, bleeding, ulceration, and loss of arm function or amputation. After multidisciplinary review, a recommendation was made to limit flow to the malformation, but to preserve future access for any potential endovascular procedures, by surgically banding the right subclavian artery at its origin - a novel approach for treatment of such a lesion. The patient’s family elected to proceed.

### Details of procedure

**Figure 4:**
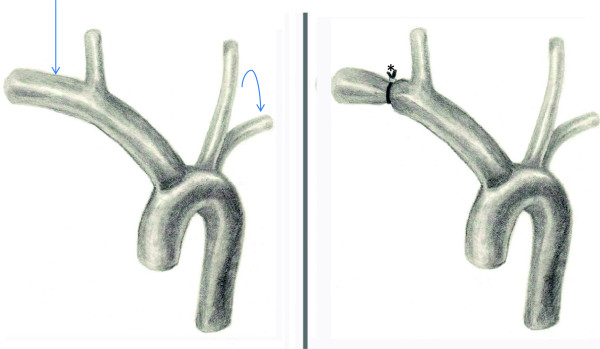
**Illustration of the surgical banding.** Note the massive dilation of the right subclavian artery (arrow) compared with left (curved arrow). The right subclavian diameter was reduced after the banding (*).

Briefly, via a median sternotomy, the pericardium was opened and a large chylous pericardial effusion was noted (triglyceride level of 1718 mg/dl). The innominate, right subclavian and right carotid arteries were exposed, taking care to protect the recurrent laryngeal nerves. A Goretex tube was passed around the proximal right subclavian artery and closed, making a band around the subclavian artery with a 3 mm lumen (Figure 4). The patient tolerated the procedure well and remained hemodynamically stable throughout.

### Follow-up

**Figure 5:**
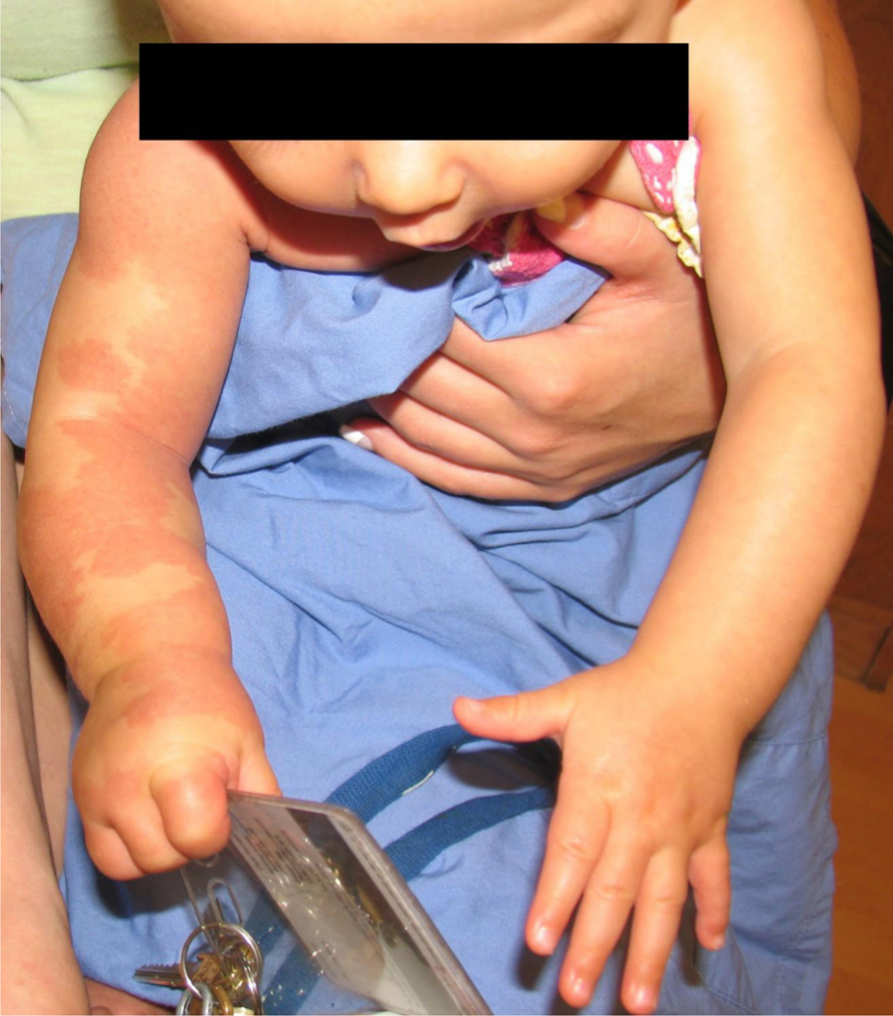
**Six months of life:** The right arm is less asymmetrically enlarged and the macular rash is less intense. The patient now demonstrates function of the upper extremity.

Immediately following the procedure, there was significant improvement in the appearance of the right arm, a finding which is maintained at six months (Figure 5). The skin lesions appeared to lighten, and the soft tissue edema progressively decreased. The patient began to use her arm, with progressive improvement. Repeat ultrasound with Doppler reflects this clinical progress, showing a trend toward decreasingly plethoric vessels and decreased edema in the soft tissues. Interestingly, the brachial artery diameter has remained unchanged (5 mm). Echocardiograms demonstrated mild and borderline right ventricular hypertrophy at one and two months postoperatively, respectively.

## Discussion

The RASA-1 gene (RAS p21 protein activator 1; OMIM 139150) located on chromosome 5q13.3 encodes the protein p120 RasGAP, a negative regulator of the Ras p21 oncogene. Mouse embryos with targeted disruptions in rasGAP fail to transform their initial aggregation of endothelial cells into a functioning vascular network [3,4]. The RASA-1 related syndromes are inherited as an autosomal dominant trait with a penetrance estimated between 89-96% [5-7].

Several families with varying inactivating mutations in the RASA-1 gene have been studied [5,6,8-12]. The phenotypic hallmark of the RASA-1 malformation appears to be the presence of multifocal capillary malformations however fast-flow vascular malformations are seen in more than one third of affected individuals. Capillary malformations in this syndrome often have a pale halo around the pink or red macular stains which is atypical for a sporadic CM [13]. The location of the associated fast-flow vascular malformations is variable. These may be identified in the intracranial space, such as a vein of Galen malformation, intradurally, or in extracranial locations, such as the face, trunk, or extremities. Visceral AVMs are not typically seen. Some patients with a RASA-1 mutation present with Parkes-Weber syndrome, characterized by multiple fast-flow vascular anomalies, including extensive intramuscular microfistula, capillary malformations, and limb overgrowth. Previously thought to be a sporadic condition, its shared genetic basis with CM-AVM suggests that the two entities are merely different manifestations of the same genetic entity. Mutations in the RASA-1 gene also underlie at least some cases of vein of Galen malformation [6]. In addition, there is evidence of defective lymphangiogenesis [14] although this is generally clinically unapparent.

The localized nature of the vascular lesions and their multifocality are intriguing in the setting of a disease caused by a germ line mutation. Some authors have invoked a somatic “second hit” mechanism to explain the large areas of unaffected tissues. Indeed, such a mechanism is reported to underlie several genetic vascular anomalies, such as glomuvenous malformation, cerebral cavernous malformation, and the PTEN mutation syndromes. Of further interest are the variable phenotypes appearing within each affected family. Our patient, for example, has a strong family history of multiple capillary malformations but without AVM or AVF. It is possible that such lesions are present and have been clinically silent, as the family members have not undergone imaging screening. However, previous work involving screened family members who carry identical mutations have documented phenotypic variability, with many genetically affected family members entirely lacking a high-flow vascular lesion. For example, this patient’s genetic alteration in the (c.2603 + 1 G > A) is identical to a patient reported by Revencu [6], et al. That patient manifested CM’s without fast-flow vascular anomalies but also was reported to have chylous ascites.

To our knowledge, this case represents the youngest reported patient diagnosed with CM-AVM and highlights the importance of early clinical detection. The presence of multiple capillary malformations should raise suspicion of a syndrome with mixed vascular anomalies. The family history and physical examination may offer important clues. Complete assessment and the broader differential diagnosis are beyond the scope of this article; however, evidence for an underlying fast-flow lesion should be sought on physical exam. This includes local warmth, palpable thrill, or an audible bruit. Any limb enlargement should be noted.

Imaging workup generally begins with ultrasound and Doppler. This case demonstrates the classic features of a fast-flow vascular malformation. There is absence of a definable mass on gray-scale ultrasound. Large, macroscopic anechoic vessels are present. There is an abnormally low resistive pattern on the spectral tracings in the feeding artery and increased pulsatility in the draining veins. We also detected a plethora of vessels, completing the assessment of an AVM. The MRI corroborated these findings.

Of interest, the ultrasound revealed abnormal echotexture and the MRI demonstrated abnormal signal within the distal musculature. Although regional edema might explain such a finding, we suspect rather that this represents numerous microfistulae in the musculature that are too small to resolve with our imaging technique. In support of this interpretation is the mild general enhancement of the involved muscles noted after gadolinium administration (Figure 3C). This underscores the overlap with PKWS. The above findings, in the context of the clinical history should strongly suggest a RASA-1 mutation.

When a significant fast-flow vascular lesion is present, a cardiac echo should also be obtained to exclude high output heart failure.

## Conclusion

We have presented what we believe to be the youngest patient to date to be diagnosed with CM-AVM syndrome. This case highlights the importance of searching for associated lesions in the presence of multifocal CM’s. When fast-flow vascular components are present, treatment can be initiated early to avoid future morbidity. A working knowledge of associated syndromes, a multidisciplinary approach and appropriate imaging studies and their interpretation can yield a timely diagnosis.

## Consent

Written informed consent for images contained in this report was obtained from the patient's parents in accordance with the guidelines of Columbia University Medical Center and the Columbia University Institutional Review Board.

## Abbreviations

CM-AVM: Capillary malformation-arteriorvenous malformation; PKWS: Parkes-Weber Syndrome; CM: Capillary malformation; AVM: Arterio-venous malformation; AVF: Arterio-venous fistula.

## Competing interests

The authors declare that they have no competing interests.

## Authors’ contributions

All authors have either written parts of, reviewed or offered changes to final manuscript, Consideration of the correct diagnosis after thorough evaluation of patient and patient follow up in team: MCG, KDM, CTL,JJK, PMM, Molecular genetic diagnosis provided by: KAY, Diagnosis suggested by imaging interpretation: GGB, PMM, Operative team and management: JJK, EB, Echocardiography and interpretation: LL, TJS, Early management and recognition of a vascular anomaly: SJK, VB, RL, Follow up of patient and contribution of figure after literature review: JC. All authors read and approved the final manuscript.
